# Gemcitabine and oxaliplatin in advanced biliary tract carcinoma: a phase II study

**DOI:** 10.1038/sj.bjc.6604628

**Published:** 2008-08-26

**Authors:** T André, J M Reyes-Vidal, L Fartoux, P Ross, M Leslie, O Rosmorduc, M R Clemens, C Louvet, N Perez, F Mehmud, W Scheithauer

**Affiliations:** 1 Department of Medical Oncology, Hôpital Tenon, Université Paris 06, Paris 75020, France; 2 Instituto Oncologico, Clinica las Condes, Santiago, Chile; 3 Department of Hepatology, Hôpital Saint-Antoine, Paris 75012, France; 4 Department of Medical Oncology, Guy's and St Thomas' Hospitals, London SE1 7EH, UK; 5 Klinikum Mutterhaus der Borromäerinnen gGmbH, Innere Medizin I, Feldstrasse 16, Trier D-54290, Germany; 6 Department of Medical Oncology, Hôpital Saint-Antoine, Paris 75012, France; 7 sanofi-aventis, Paris 75601, France; 8 Department of Internal Medicine 1 and Cancer Center, Medical University, Vienna A-1090, Austria

**Keywords:** biliary tract carcinoma, gemcitabine, GEMOX, oxaliplatin

## Abstract

Advanced biliary tract carcinomas (BTCs) are often diagnosed at an advanced/metastatic stage and have a poor prognosis. The combination of gemcitabine and oxaliplatin (GEMOX) has shown promising activity in this setting. This international phase II study evaluated the efficacy and safety of GEMOX as first-line therapy in patients with advanced BTCs. Eligible patients with previously untreated locally advanced or metastatic BTC received gemcitabine 1000 mg m^−2^ (day 1) and oxaliplatin 100 mg m^−2^ (day 2), every 2 weeks. Seventy patients were enroled; 72.9% had metastatic disease. Sixty-seven patients were treated. There were 10 confirmed partial responses (14.9%; 95% confidence interval (CI), 7.4–25.7%) in the treated population (RECIST). Twenty-four patients (35.8 %) had stable disease. The objective response rate was 20.5% in patients with non-gallbladder cancers (9/44 patients) and 4.3% in patients with gallbladder cancers (1/23). Median overall survival for the intent-to-treat population was 8.8 months (95% CI, 6.9–11.1%) and progression-free survival was 3.4 months (95% CI, 2.5–4.6%). Grade 3/4 toxicities included thrombocytopenia (14.9% of patients), alanine aminotransferase elevation (13.4%), anaemia (10.4%), neutropenia (11.9%) and pain (11.9%). In this study, GEMOX demonstrated activity in non-gallbladder carcinoma, but poor activity in gallbladder carcinoma. GEMOX is well tolerated in advanced BTCs.

Biliary tract carcinomas (BTCs), comprising gallbladder carcinoma (GBC) and intra- and extrahepatic cholangiocarcinoma (CC), are relatively rare in the United States and Europe (de Groen *et al*, 1999). For example, approximately 5000 cases of GBC and 2000–3000 cases of CC are diagnosed annually in the United States. There are marked geographical variations in the incidence of GBC, although it is consistently more common in women than in men (Lazcano-Ponce *et al*, 2001). The symptoms of BTC are non-specific and tumours have often reached an advanced stage at diagnosis. As such, the prognosis for patients with BTC is extremely poor: median survival is generally lesser than 6 months and estimated 1- and 2-year survival rates are 25 and 13%, respectively (Patel, 2002). Chemotherapy is a palliative treatment option for patients with advanced disease. Owing to the lack of randomised phase III studies, there is no standard chemotherapy for advanced BTC. One clinical trial has demonstrated the improved survival for chemotherapy (5-fluorouracil plus leucovorin with or without etoposide) *vs* best supportive care (Glimelius *et al*, 1996), although the ability of chemotherapy to prolong survival remains to be confirmed.

Tolerability is of major importance when selecting palliative treatment regimens. Gemcitabine has palliative benefits and is generally well tolerated as therapy for advanced pancreatic carcinoma (Heinemann, 2002). Gemcitabine is also widely used as palliative therapy for advanced BTCs because of histogenetic similarities between the pancreas and biliary tract (Scheithauer, 2002).

Gemcitabine has shown promising activity against advanced BTCs, with response rates (RRs) in the range of 12–35% when used in combination with agents such as 5-fluorouracil, cisplatin, mitomycin C, or capecitabine (Kornek *et al*, 2004; Alberts *et al*, 2005; Kim *et al*, 2006; Riechelmann *et al*, 2007; Lee *et al*, 2008). A recent randomised phase II study suggested that combination chemotherapy with gemcitabine and cisplatin may be more effective than gemcitabine alone (Valle *et al*, 2006). The overall RR (ORR) was 24.3% for combination therapy and 15.2% for gemcitabine alone (complete response (CR)+partial response (PR)+stable disease (SD) was 75.7 *vs* 57.6%, respectively). Time to progression was also longer in the combination group (8.0 months) than in the monotherapy group (5.5 months). A follow-up study has been initiated with adequate power to assess the potential survival benefit of adding cisplatin to gemcitabine.

Oxaliplatin was used as monotherapy in one phase II study as first-line treatment for patients with BTC. An objective RR of 20.6% was observed with an overall survival (OS) of 7 months (Androulakis *et al*, 2006). Preclinical studies have demonstrated antitumour activity for the combination of gemcitabine and oxaliplatin (GEMOX) in human leukaemia and colorectal cancer cell lines and provide the rationale for using this combination in clinical studies. An optimal sequence-dependent synergy is apparent, with exposure to gemcitabine first and oxaliplatin later (Faivre *et al*, 1999).

A French phase II study (conducted in two centres) showed that the GEMOX combination was active and well tolerated as first-line chemotherapy in 36 patients with advanced BTCs (André *et al*, 2004); ORR (without confirmed response for all patients) was 35.5% (95% confidence interval (CI), 18.7–52.3%), progression-free survival (PFS) was 5.7 months and OS was 15.4 months. We undertook the present international phase II study to evaluate the efficacy and tolerability of GEMOX as first-line chemotherapy in a larger group of patients with advanced BTCs.

## Patients and methods

### Eligibility criteria

Patients aged >18 years with histologically proven, locally advanced or metastatic carcinoma of the biliary tract (gallbladder, intrahepatic bile ducts, extrahepatic bile ducts and ampulla of Vater) were enroled in the study. Other eligibility criteria included: Eastern Cooperative Oncology Group performance status ⩽2; unidimensionally measurable disease (Therasse *et al*, 2000); no prior chemotherapy for advanced disease; and adequate haematological (absolute neutrophil count >1.5 × 10^9^ l^−1^, platelets >100 × 10^9^ l^−1^), renal (creatinine <1.5 × the upper limit of normal; ULN), and hepatic function (alanine aminotransferase <5 × ULN; bilirubin <2.5 × ULN). Patients with jaundice or evidence of bile duct obstruction and in whom the biliary tree could be decompressed by endoscopic percutaneous endoprosthesis, with a subsequent reduction in bilirubin to <2.5 × ULN, were also eligible.

Patients with prior malignancy or prior chemotherapy for advanced disease, central nervous system metastases or peripheral neuropathy grade ⩾2 were excluded from the study. Prior radiation therapy within 4 weeks of the first gemcitabine administration was not permitted. Women of childbearing potential were required to be neither pregnant nor breastfeeding and to be under active contraception.

The study was conducted in compliance with Good Clinical Practice. All patients provided written informed consent.

### Treatment plan

Treatment consisted of gemcitabine 1000 mg m^−2^ as a 100-min i.v. infusion on day 1 followed by oxaliplatin 100 mg m^−2^ as a 2-h i.v. infusion on day 2. Cycles were repeated every 2 weeks. Treatment was continued until disease progression, unacceptable toxicity, patient withdrawal of consent, or treatment delay of more than 3 weeks.

Oxaliplatin was stopped altogether for grade 3 neurological symptoms but could be reintroduced upon recovery (to grade ⩽2). For paraesthesia without pain that persisted between cycles (lasting greater than 14 days), oxaliplatin was stopped until recovery, and then restarted at 75 mg m^−2^. For paraesthesia with pain or functional impairment that persisted for greater than 7 but fewer than 14 days, the dose of oxaliplatin was reduced to 75 mg m^−2^; if the pain or functional impairment persisted between cycles, oxaliplatin was stopped. In the event of pharyngolaryngeal dysaesthesia during an infusion, the duration of oxaliplatin infusion was extended to 6 h for that and for subsequent infusions.

For patients with a platelet count <75 × 10^9^ l^−1^, treatment was delayed for up to 3 weeks to allow recovery, and the oxaliplatin dose was reduced by 15% in subsequent cycles. For grade 3/4 thrombocytopenia, neutropenia, mucositis, diarrhoea or asthenia, the dose of gemcitabine was reduced to 800 mg m^−2^ over 80 min and the dose of oxaliplatin was reduced to 85 mg m^−2^ over 2 h. If grade 3/4 toxicity developed after dose reduction, the patient could be withdrawn from the study. In the event of oxaliplatin discontinuation for any toxicity, gemcitabine could be continued at the same dose and same schedule (every 2 weeks).

### Assessments and follow-up

Tumour response was evaluated after four treatment cycles and every four cycles thereafter using the Response Evaluation Criteria in Solid Tumours (RECIST) unidimensional criteria (Therasse *et al*, 2000). Objective responses were to be confirmed after at least 4 weeks. Tumour burden was assessed in both target and non-target lesions. Target lesions were defined as lesions that were measurable in at least one dimension, with the longest diameter ⩾20 mm using conventional techniques or the longest diameter ⩾10 mm measured using spiral computed tomographic (CT) scan. Computed tomographic and magnetic resonance imaging scans were the only accepted imaging techniques for the determination of response. Non-target lesions were defined as all other lesions and non-measurable lesions. The integrated response was derived from the investigator's assessment of target and non-target lesions, taking into account the appearance of new lesions in accordance with RECIST. For target lesions, SD was categorised as neither sufficient shrinkage to qualify for PR nor sufficient increase to qualify for progressive disease (PD), taking the the smallest sum of the longest diameter as reference since the treatment started; for non-target lesions, SD was categorised as neither CR or PD. Assignment to the SD category could only be made at least 6 weeks (two cycles) after the start of treatment. The best overall response was determined following a sequential review of all integrated responses recorded from the start of treatment until disease progression, with the last evaluation taken as the 30-day post-treatment follow-up. Sequential evaluations were compared, and the best response from each pair was compared with the subsequent pair to derive the best overall response.

For patients who discontinued treatment for reasons other than disease progression, tumour evaluations were performed every 2 months. Whenever possible, the follow-up was continued until death.

Safety evaluations were performed in the exposed population before administration of each cycle and toxicity was graded using National Cancer Institute Common Toxicity Criteria version 2.

### End points and statistics

The primary objective of this study was to evaluate the efficacy (based on RR) of the GEMOX regimen as first-line therapy in patients with advanced BTCs and rejecting the treatment for further study, if it was found to be insufficiently active. The study employed a one-stage design (Fleming, 1982) incorporating the following assumptions: H0: RR ⩽20% and H1: RR ⩾35%. On the assumption that of 56 evaluable patients, using a significance level of 0.05 and a power of 80%, GEMOX would be declared insufficiently active if ⩽12 responses were observed, and declared active if >12 responses were observed. To be evaluable for response, patients were to receive a minimum of two cycles of GEMOX (ie, 6 weeks on study) and were to have had at least one post-baseline tumour assessment unless early progression occurred first, in which case, patients were considered evaluable for response. Assuming that 25% of patients would have non-measurable disease, a sample of 70 patients was required, so that a total of 56 patients attained the requirements of the evaluable patient population.

An exploratory analysis was conducted to evaluate RRs by tumour type (GBC and CC). Kaplan–Meier survival curves were computed for time-to-event variables. Progression-free survival was calculated from the date of first treatment administration to the earliest date of disease progression, death or data cutoff. Overall survival was calculated from the date of first treatment administration to the date of death. Safety was reported for all subjects who received at least one dose of study drug.

## Results

### Patient characteristics

Between April 2003 and April 2005, 70 patients were enroled in the study, which was conducted in seven centres in France (2), Germany (2), Austria (1), Chile (1) and the United Kingdom (1). Patient and tumour characteristics at baseline are shown in Table 1.

### Treatment received

Three patients did not receive study treatment: two died before starting treatment (one with GBC, one with CC) and one patient with CC had hyperbilirubinaemia. The exposed population, therefore, comprised 67 patients. In total, 479 cycles of oxaliplatin and 521 cycles of gemcitabine were administered. The median number of cycles was five for both oxaliplatin (range: 1–18 cycles) and gemcitabine (range: 1–30 cycles). Mean relative dose intensities were 94.1% for oxaliplatin and 98.4% for gemcitabine in the exposed population.

The primary reason for treatment discontinuation was disease progression (48 patients; 68.6%). Ten patients (14.3%) discontinued treatment as a result of adverse events (AEs), which were considered to be oxaliplatin-related hypersensitivity reactions in three patients (4.3%). Five patients continued treatment with gemcitabine alone after discontinuing oxaliplatin.

### Efficacy

There were 10 PRs (14.9%;. 95% CI, 7.4–25.7%) in the exposed population (Table 2). A further five unconfirmed PRs were observed in the exposed population (three GBCs and three CCs). The majority of responses were observed in patients with CC: PRs were observed for 9/44 patients (20.5%) with CC and 1/23 patients (4.3%) with GBC.

**Figure 1:**
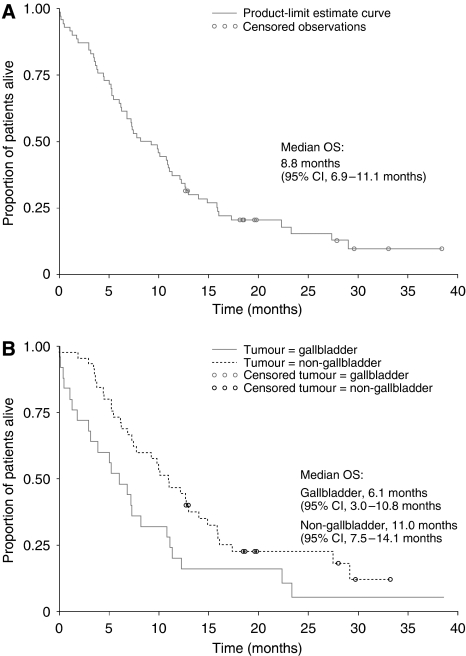
Progression-free survival (**A**) for the intent-to-treat population (*n*=70) and the (**B**) subgroup analysis of patients with gallbladder (*n*=25) and non-gallbladder (*n*=45) tumours.

Median PFS was 3.4 months (95% CI, 2.5–4.6 months) for both the ITT and exposed populations (Figure 1A). Median PFS was 2.5 months for patients with GBC (95% CI, 1.6–4.3 months) and 3.8 months for patients with CC (95% CI, 2.7–5.6 months; Figure 1B).

**Figure 2:**
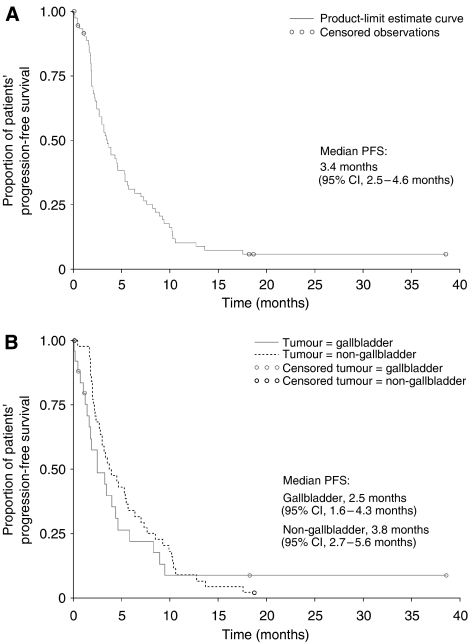
Overall survival for the (**A**) Intent-to-treat population (*n*=70) and the (**B**) subgroup analysis of patients with gallbladder (*n*=25) and non-gallbladder (*n*=45) tumours.

Overall, 59 deaths were reported during the study period in the ITT population, 57 of which were in the exposed population. One patient died 1 month after the study cutoff date, but was considered alive for the purpose of the survival analysis. Median OS was 8.8 months (95% CI, 6.9–11.1 months) in the ITT population and 9.3 months (95% CI, 6.9–11.4 months) in the exposed population (Figure 2A). For both populations, median OS was 11.0 months for patients with non-GBC and 6.1 months for patients with GBC (Figure 2B).

### Safety

Sixty-seven patients received at least one administration of study treatment and were included in the safety analysis. There were six deaths during the treatment period (between cycle 1 and cycle 4), none of which was considered by the investigators to be treatment-related. However, after reviewing the patient data, the sponsor could not rule out a relationship to study treatment for three deaths; one patient developed septicaemia and had global status deterioration, one patient had diarrhoea and vomiting leading to the rapid health deterioration and respiratory arrest, and one patient died from general health deterioration.

Overall, nausea (82.1%) and vomiting (56.7%) of all grades were frequent side effects, despite systemic prophylactic measures (Table 3). Grade 3 nausea and vomiting occurred in 4.5% and 10.4% of patients, respectively, although there were no grade 4 events of this type. Overall, grade 3/4 AEs occurred in 47 patients (70.1%).

Peripheral sensory neuropathies were observed in 67.2% of patients, with grade 3 neuropathy in 6.0%, including one case of laryngospasm. No grade 4 neuropathy was reported. In general, neuropathies were mild (grade 1) and intermittent during the first treatment cycles but tended to worsen and become more persistent as the number of treatment cycles increased. Neurotoxicity grade ⩾2 occurred in 40% of patients who received ⩾9 treatment cycles. Other frequently reported AEs included anaemia (77.6%), fatigue (73.1%), thrombocytopenia (68.7%), liver enzyme increase (62.7%), and weight loss (61.2%), although the majority of these events were grade 1/2 in severity.

## Discussion

This study is one among the largest of the phase II studies conducted to date to evaluate the efficacy and tolerability of chemotherapy in patients with advanced BTCs. The majority of patients enroled in this study had metastatic disease. The GEMOX administered every 2 weeks was well tolerated and conferred an ORR of 14.9%, with 50.7 % of patients achieving a PR or SD.

The ORR is somewhat lower than previously reported for GEMOX (André *et al*, 2004) or for gemcitabine in combination with other chemotherapy agents (Kim *et al*, 2006; Eckel and Schmid, 2007; Riechelmann *et al*, 2007; Lee *et al*, 2008). Our goal of achieving a 20% RR in this phase II study was not reached, when responses were assessed using RECIST. However, the percentage of combined confirmed and unconfirmed PR (23.9%) was more in line with other published phase II studies in advanced BTCs, the majority of which report RRs that are often unconfirmed, as opposed to using RECIST confirmed responses. In our study, most unconfirmed PRs were those that did not persist from one assessment to the next (tumour response evaluation every 2 months).

There is currently no standard chemotherapy regimen for advanced BTCs. Gemcitabine is one of the most widely used agents in this setting, with RRs of 12–35% for gemcitabine-based combination regimens, although these rates are unconfirmed in the majority of studies. A pooled analysis of studies (112 studies, including one phase III study; 2810 patients) performed between 1985 and 2006 demonstrated that the combination of gemcitabine with platinum compounds increased RRs and tumour-control rates compared with gemcitabine alone (Eckel and Schmid, 2007). Response rates of 22–50% have been observed in single-institution studies, with OS of 7.6–15.4 months (André *et al*, 2004; Harder *et al*, 2006; Verderame *et al*, 2006). However, single-institution studies often report higher RRs than multinational studies.

For this study, we conducted an experimental subgroup analysis to assess response to treatment in patients with GBC and CC. Our study population comprised approximately one-third of the patients with GBC, who are generally considered to have a worse prognosis than patients with other BTCs (Gallardo *et al*, 2005). In BTC, prognostic factors could be more important than therapy itself in determining treatment outcome. This study confirms the differential outcome between the gallbladder compared with other cancers of the biliary tree, as observed by Gallardo *et al* (2005).

In the pooled analysis by Eckel and Schmid (2007), the median RR for trials in patients with GBC was higher than that in patients with CC (35.5 *vs* 17.7%; *P*=0.008). However, the response duration is short for GBC, and OS was significantly longer in trials of patients with CC compared with GBC (median 9.3 *vs* 7.2 months; *P*=0.048) (Eckel and Schmid, 2007). In our study, RRs and RR+SD values in the GBC and CC subgroups were 4.0 *vs* 20.0% and 40 *vs* 53%, respectively. Concerning the survival, GBC was associated with a shorter PFS duration than CC (2.5 *vs* 3.8 months, respectively) and shorter median OS (6.1 *vs* 11.0 months, respectively). The reported efficacy,especially for GBC, differed markedly from a previous study of GEMOX in BTC, where the RR (unconfirmed) and OS were higher for GBC (*n*=11) *vs* CC (*n*=16; 54.4 *vs* 21.4%; 16.0 *vs* 14.5 months; André *et al*, 2004). The differences in the results of the two studies are surprising and could be related to the small number of patients, a centre effect, or the inclusion of unconfirmed responses.

The combination of gemcitabine and oxaliplatin was generally well tolerated. Grade 3/4 AEs were reported in 70.1% of patients, although all patients experienced at least one AE of any grade. As previously observed with the GEMOX combination (André *et al*, 2004), the most frequent grade 3/4 AEs were neutropenia, thrombocytopenia, pain and vomiting, all of which occurred in <15% of patients. Neutropenia and thrombocytopenia appear to occur less frequently with the GEMOX regimen than with the combination of gemcitabine and cisplatin (Park *et al*, 2006; Lee *et al*, 2008; Meyerhardt *et al*, 2008). Patients with advanced BTCs are very susceptible to infection and disease progression. Therefore, caution will be necessary with GEMOX chemotherapy like all chemotherapies in this disease. Grade 3 sensory neuropathy was uncommon (6.0%).

In conclusion, this multinational study provides further evidence for the activity of GEMOX as a treatment for non-GBC, but also demonstrates the poor activity of this agent in GBCs. The combination of gemcitabine and oxaliplatin is well tolerated and provides a treatment option for patients with advanced BTCs, and in particular non-GBCs. A phase III study comparing GEMOX to gemcitabine is necessary to further establish the role of GEMOX in advanced BTCs. The design of such a study should include stratification for the location of the carcinoma (non-GBCs *vs* GBCs).

## Figures and Tables

**Table 1:** Patient and tumour characteristics at baseline (intent-to-treat population)

	**Tumour type**		
**Characteristics**	**Non-gallbladder (*n*=45)**	**Gallbladder (*n*=25)**	**All (*n*=70)**
*Gender,* no. *(%)*			
Male	22 (48.9)	6 (24.0)	28 (40.0)
Female	23 (51.1)	19 (76.0)	42 (60.0)
Median age, years (range)	62 (30–83)	55 (30–72)	62 (30–83)
			
*ECOG PS,* *no.* *(%)*			
0	23 (51.1)	12 (48.0)	35 (50.0)
1	21 (46.7)	10 (40.0)	31 (44.3)
2	1 (2.2)	3 (12.0)	4 (5.7)
			
*Primary tumour location,* *no.* *(%)*			
Gallbladder	0 (0.0)	25 (100.0)	25 (35.7)
Intrahepatic bile ducts	30 (66.7)	0 (0.0)	30 (42.9)
Extrahepatic bile ducts	13 (28.9)	0 (0.0)	13 (18.6)
Intra/extrahepatic bile ducts	1 (2.2)	0 (0.0)	1 (1.4)
Ampulla of Vater	1 (2.2)	0 (0.0)	1 (1.4)
			
*Prior treatment for BTC,* *no.* *(%)*			
Surgery	18 (40.0)	20 (80.0)	38 (54.3)
Radiotherapy	0 (0.0)	1 (4.0)	1 (1.4)
			
*Disease status,* *no.* *(%)*			
Metastatic	32 (71.1)	19 (76.0)	51 (72.9)
Locally advanced	13 (28.9)	6 (24.0)	19 (27.1)

BTC=biliary tract carcinoma; ECOG PS=Eastern Cooperative Oncology Group performance status.

**Table 2:** Best overall response to treatment by RECIST (exposed population)

**Response, *n* (%)**	**Non-gallbladder (*n*=44)**	**Gallbladder (*n*=23)**	**Total (*n*=67)**
CR	0 (0)	0 (0.0)	0 (0.0)
Confirmed PR	9 (20.5)	1 (4.3)	10 (14.9)
SD	15 (34.1)	9 (39.1)	24 (35.8)
PD	17 (38.6)	10 (43.5)	27 (40.3)^a^
Not assessable	3 (6.8)	3 (13.0)	6 (9.0)
Overall tumour control rate (CR+PR+SD)	24 (54.5)	10 (43.5)	34 (50.7)

CR=complete response; PD=progressive disease; PR=partial response; RECIST=Response Evaluation Criteria in Solid Tumors; SD=stable disease.

a For the intent-to-treat analysis, three patients who were not exposed to treatment were considered to have PD: two died before starting treatment (gallbladder carcinoma, one patient; cholangiocarcinoma, one patient) and one patient with cholangiocarcinoma had hyperbilirubinaemia.

**Table 3:** Main National Cancer Institute Common Toxicity Criteria adverse events (exposed population)

	**Number of patients (%) (*n*=67)**		
**Adverse events**	**All grades 1–4**	**Grade 3**	**Grade 4**
*Haematological*			
Anaemia	52 (77.6)	5 (7.5)	2 (3.0)
Thrombocytopenia	46 (68.7)	9 (13.4)	1 (1.5)
Neutropenia	26 (38.8)	5 (7.5)	3 (4.5)
Neutropenic infection	2 (3.0)	2 (3.0)	0 (0.0)
Febrile neutropenia	1 (1.5)	1 (1.5)	0 (0.0)
			
*Non-haematological*			
Alanine aminotransferase increase	42 (62.7)	8 (11.9)	1 (1.5)
Aspartate aminotransferase increase	1 (1.5)	0 (0.0)	0 (0.0)
Hyperbilirubinaemia	3 (4.5)	3 (4.5)	0 (0.0)
Nausea	55 (82.1)	3 (4.5)	0 (0.0)
Vomiting	38 (56.7)	7 (10.4)	0 (0.0)
Weight loss	41 (61.2)	3 (4.5)	0 (0.0)
Fatigue	49 (73.1)	4 (6.0)	3 (4.5)
Peripheral sensory neuropathy	45 (67.2)	4 (6.0)	0 (0.0)
Pain	40 (59.7)	8 (11.9)	0 (0.0)
Infection	14 (20.9)	5 (7.5)	0 (0.0)
Thrombosis	3 (4.5)	2 (3.0)	1 (1.5)
